# Supplementary figures and images for: A mechanistic model of snakebite as a zoonosis: Envenoming incidence is driven by snake ecology, socioeconomics and its impacts on snakes
Source: PLoS Negl Trop Dis. 2022 May 12;16(5):e0009867. doi: 10.1371/journal.pntd.0009867 (PMC9129040; doi:10.1371/journal.pntd.0009867)

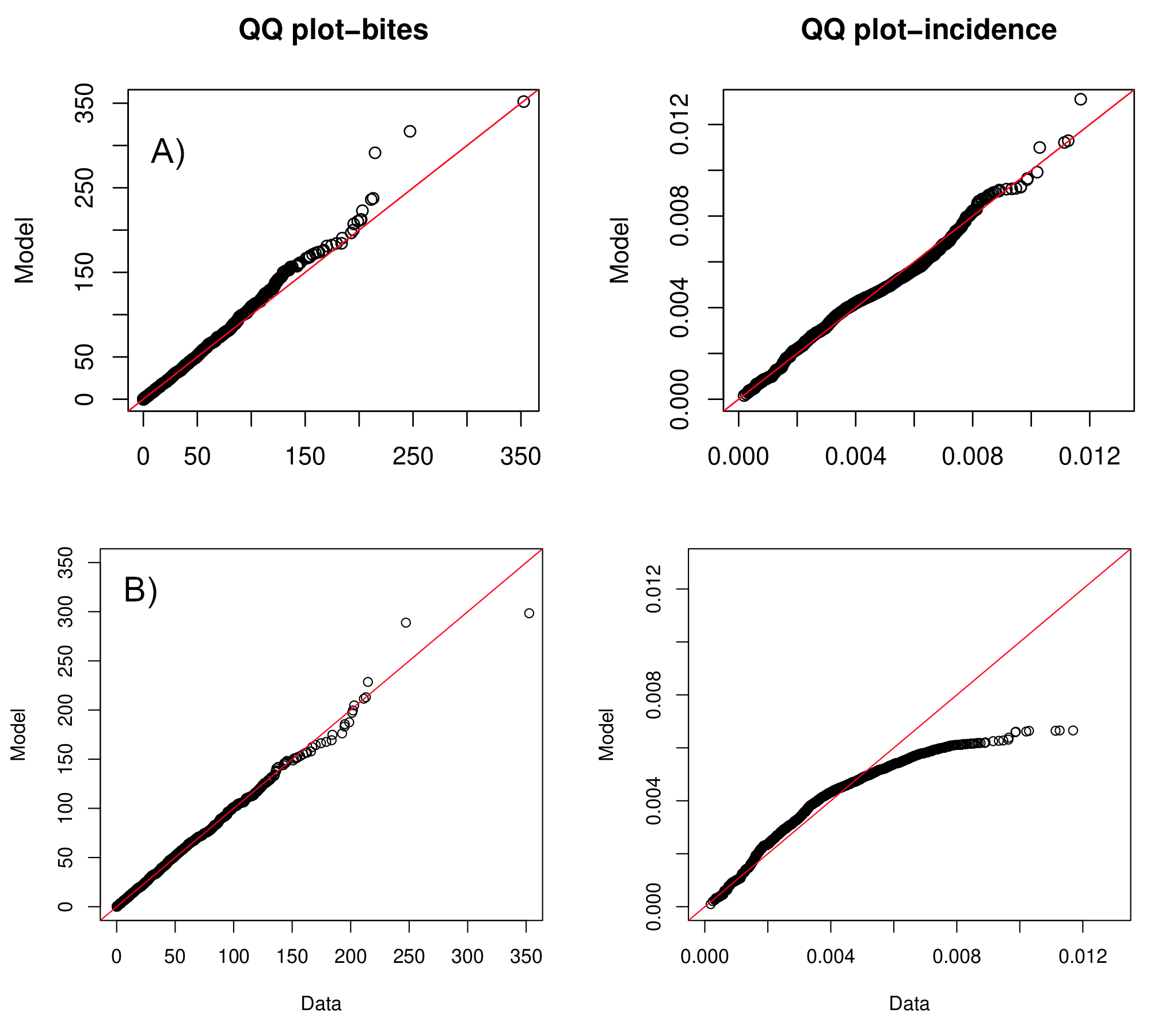

Supplement: S1 Fig — A) Mass-action model and B) refuge effect model. The curved shape of incidence rates for the refuge effect model indicates that the distribution of incidence was very different from the incidence rates used as data. (TIF) [file pntd.0009867.s001.tif]

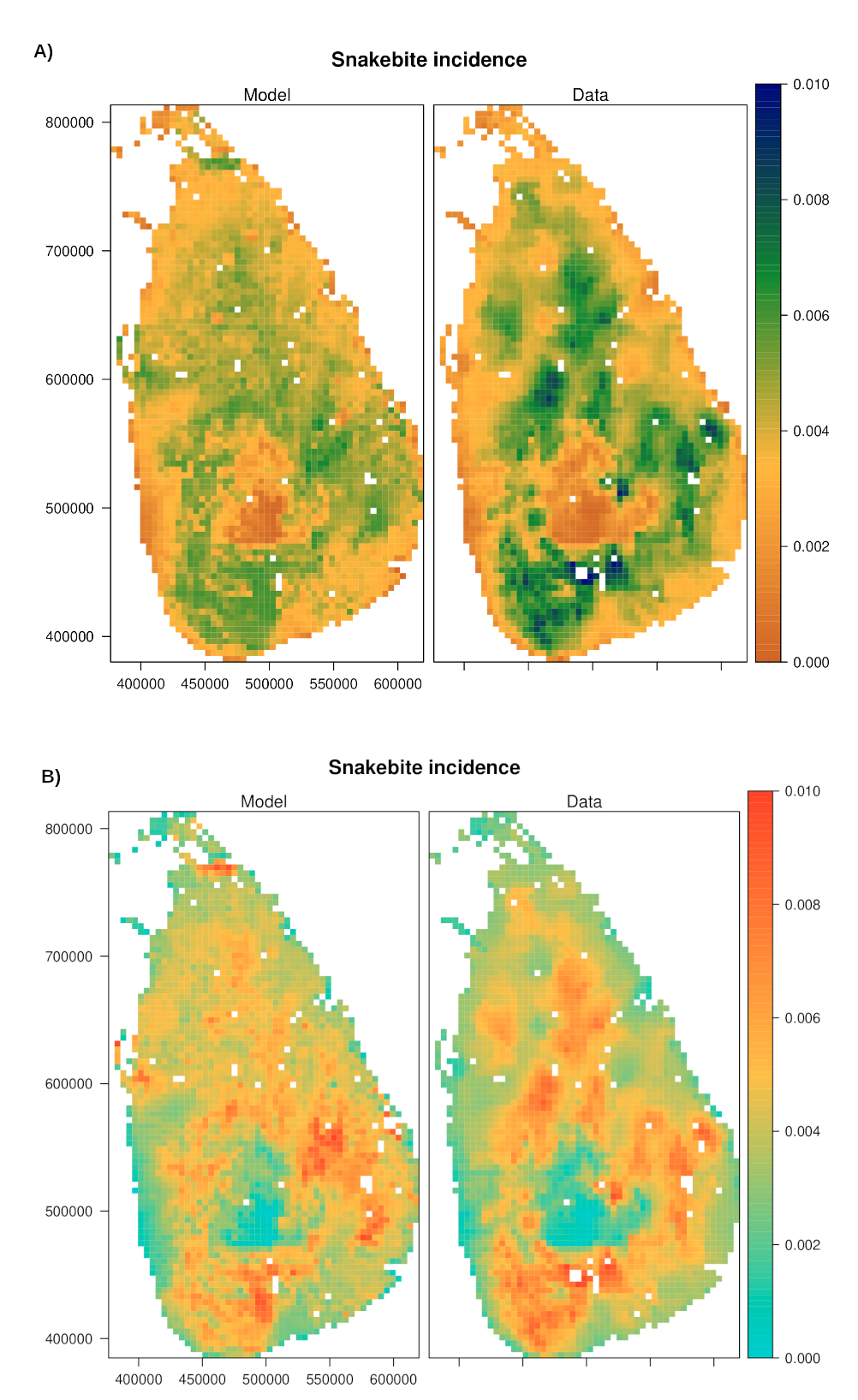

Supplement: S2 Fig — A) Refuge effect and B) Mass action. (TIF) [file pntd.0009867.s002.tif]

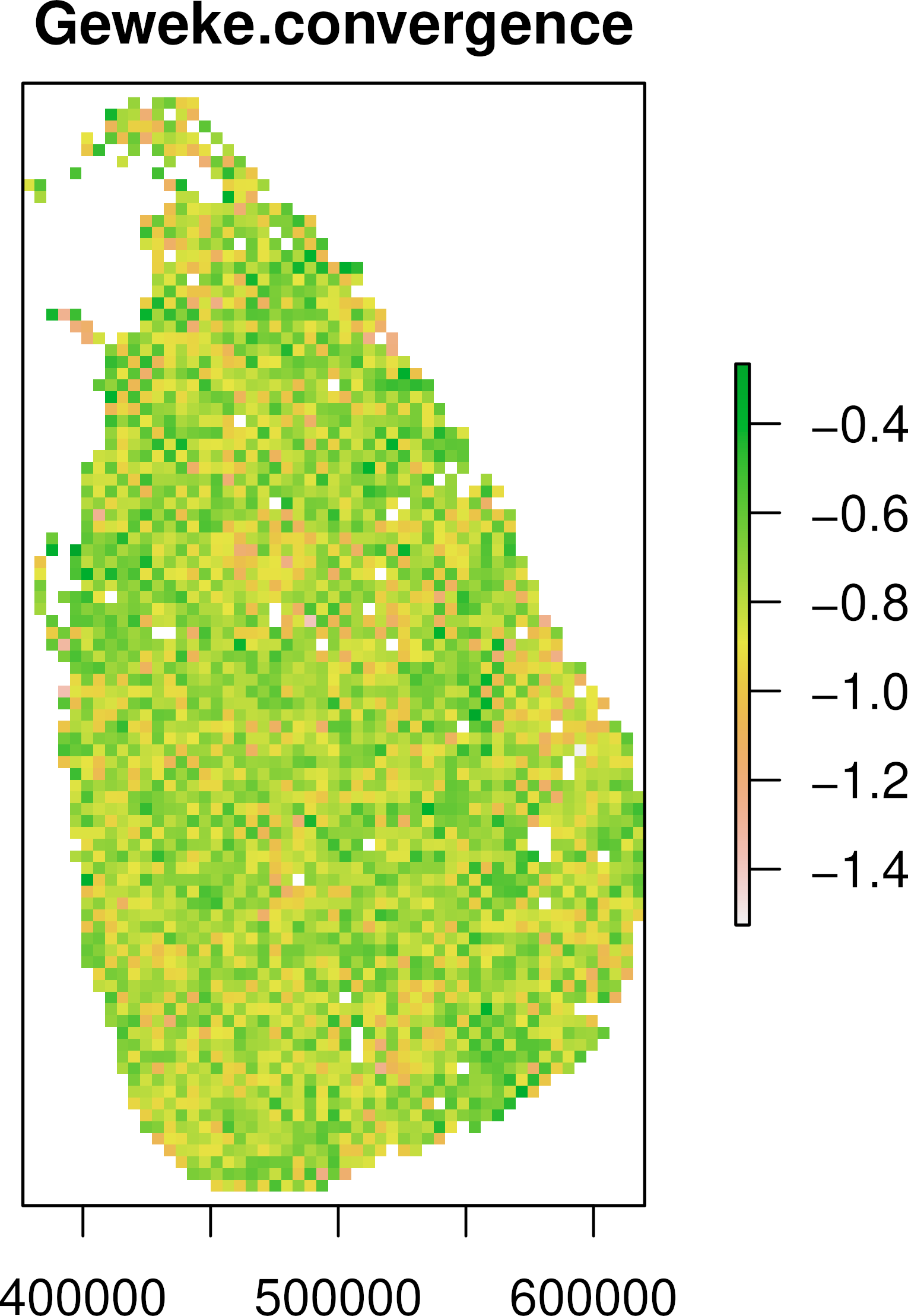

Supplement: S3 Fig — Estimates are expected to lie within -2 and 2 for sampling convergence. (TIF) [file pntd.0009867.s003.tif]

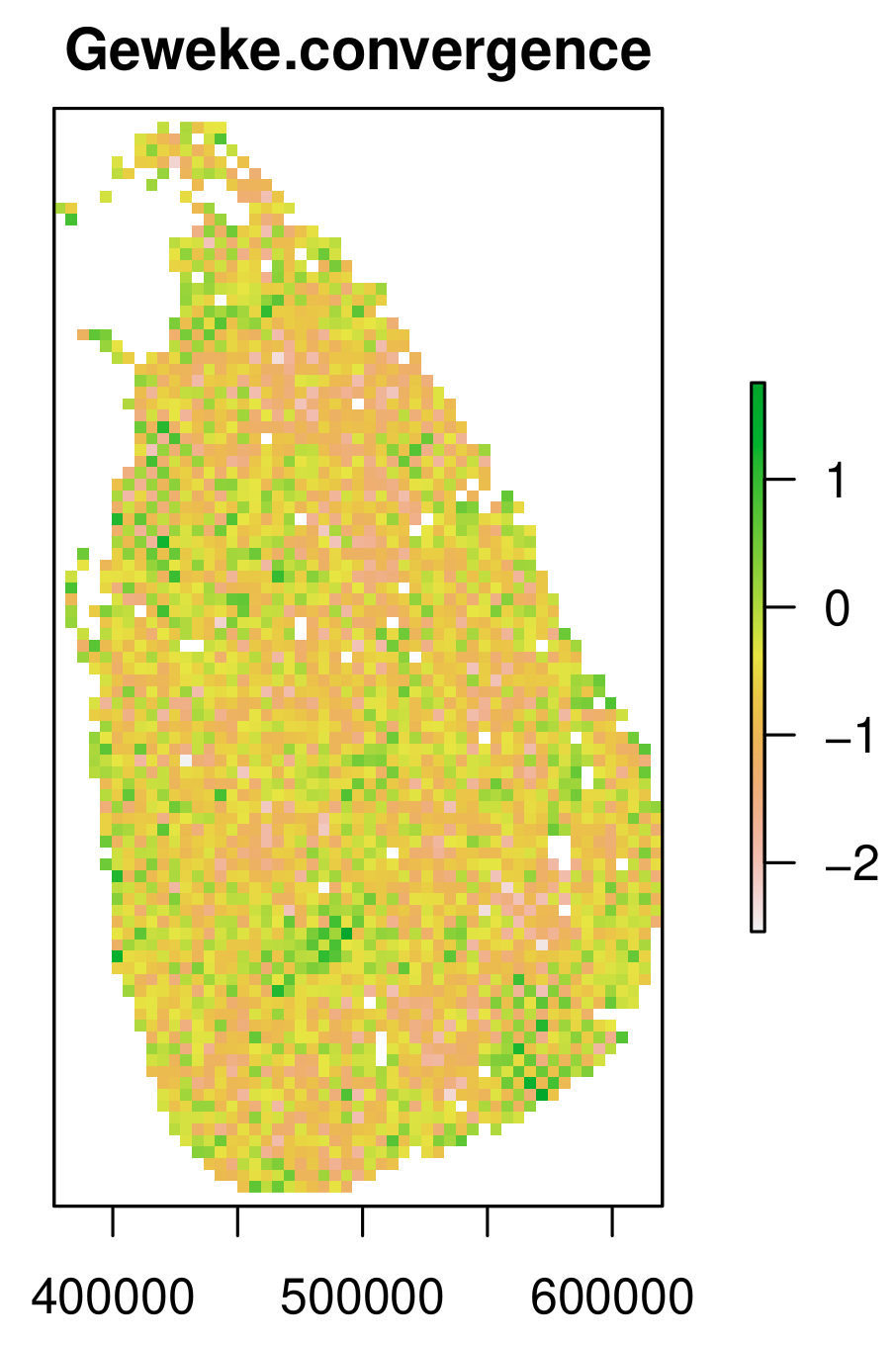

Supplement: S4 Fig — Estimates are expected to lie within -2 and 2 for sampling convergence. (TIF) [file pntd.0009867.s004.tif]
