# Supplementary material for: A mechanistic model of snakebite as a zoonosis: Envenoming incidence is driven by snake ecology, socioeconomics and its impacts on snakes
Source: PLoS Negl Trop Dis. 2022 May 12;16(5):e0009867. doi: 10.1371/journal.pntd.0009867 (PMC9129040; doi:10.1371/journal.pntd.0009867)
Supplement: Attachment — Submitted filename: Response-reviewers.pdf [file pntd.0009867.s008.pdf]

Response to reviewer’s comments

We thank the opportunity to respond to reviewer’s comments, and believe that these considerably improve the quality of the manuscript. We found that many of the comments made reference to the same aspects, in such instances hence we refer in our responses to the changes made earlier in this same document. For guidance, all reviewer comments appear in grey, our responses and explanations are preceded by “**R:**” and quotations from the main manuscript appear in *italics*, where bold ***italics highlight*** insertions.

Methods

Reviewer #1

The title of the study "Redefining snakebite envenoming as a zoonosis: disease incidence is driven by snake ecology, socioeconomics and anthropogenic impacts" is not perfectly matching or at least some sort of misleading to what the method/object/design of this study is.

The manuscript itself deals more with the application of epidemiological models of zoonosis on snakebite data in a certain region, while the title somehow suggests a more fundamental attempt/hypothesis/discussion: whether snakebite is a zoonosis.

R: Thank you for suggestions. We followed the advice and proposed the following new title:

A mechanistic model of snakebite as a zoonosis: envenoming incidence is driven by snake ecology, socioeconomics and its impacts on snakes

Regrouping snakebite as a zoonosis would of course broaden the general interest and attention to this devastating neglected disease, what would be highly desirable. Nevertheless, the definition of a zoonosis stated by the WHO is: "A zoonosis is any disease or infection that is naturally transmissible from vertebrate animals to humans" and "A zoonosis is an infectious disease that has jumped from a non-human animal to humans. Zoonotic pathogens may be bacterial, viral or parasitic, or may involve unconventional agents and can spread to humans through direct contact or through food, water or the environment." (https://www.who.int/news-room/fact-sheets/detail/zoonoses). Snakebite or snake venom neither matches well the medical definition of infection/infectious nor exactly acts as a "transmitted"/"jumping" disease, because it has not been a disease/pathogen to the snake.

R: Thank you for this insight. While our original intent was to show that the pathogenic agent in this case is a chemical substance (the venom), we totally agree, and have followed the recommendations and changed the title accordingly (see change above).

If snakebite would be suggested as a zoonosis(hypothesis), this would definitely need more discussion here. Additionally, the sheer applicability of epidemiological models used for zoonosis on snakebites is not a proof of snakebite being a zoonosis itself. These zoonosis models roughly describe the probability of a potentially dangerous vertebrate animal- human encounter, highly differentiated

towards socioecological factors etc.. These models can be adapted to lot of non-zoonotic infectious diseases, like (non-vertebrate-animal-) vector borne diseases. If we accept that, like the snake venom, the threat of the animal to the human is not a disease but rather a inherent property/ability of the animal, we can adapt such zoonosis models to a lot of incidents as fatalities by hippopotamus or animal-inflicted car accidents (transmission of physical force/energy), and if we delete the „vertebrate" we can probably even use a "zoonosis" model for further animal-human encounters as arachnidae/insect stings or lepidopterism.

R: Thank you for your insights. We agree, that mass action in mathematical terms does not justify the description of a non-infectious disease with a term that describes some infectious diseases, and have changed the title accordingly (see above).

Reviewer #2

The author used appropriate mathematical formulas to represent zoonotic spillovers for the snakebites in Sri Lanka, which is the hotspot of snakebite envenoming. Several formulas were used to best represent the burden attributed to the association between reservoirs (i.e., snake distribution in their habitat, their nature), their spillover hosts (human density/occupation) and locations. Therefore, the study is appropriately designed to explain the mechanistic eco epidemiological aspects and understanding of snakebite risks relating to demographic data.

To support the hypothesis, the author used previous research to calculate the mathematical formulas for the distribution and abundancy of reservoir hosts (snakes) and spillover hosts (humans). With different methods formulated depending on the condition of snakes and humans, the study is well-articulated to the objectives.

R: Thank you.

Reviewer #3

Accept

R: Thank you

Reviewer #4

Snakebite to be considered a zoonotic disease is a very important study and perspective. The only concern is the data being referred is unclear and more than a decade old. …

R: Thank you. The dataset may not be the latest today, but it was the only available data which permitted our analyses when our study was conducted. Other studies that estimate snakebite incidence lack the spatial resolution and extent necessary for our analyses.

Urbanization, deforestation, increase in population and climate change related untimely season changes have caused environmental changes affecting flora and fauna in many places. Urbanization juxtaposing with farming activities and irresponsible garbage dumping seem to have resulted in a higher number of man-animal conflict cases in countries in Southeast Asia, especially where snake distribution thrives on prey base found abundantly close to human habitations.

R: Indeed environmental degradation and its implications for populations of venomous snakes are important. All of these factors are indirectly accounted for in our model via land cover and estimates of potential snake abundance (lines 138-140, Methods). Explicitly accounting for the effect of the mentioned dynamic processes on snakebite at the spatial scale of our study is still impossible, mostly due to data availability. To make this clear, we have modified lines 439-441 in the discussion:

"*All of these characteristics give our model unparalleled forecasting capabilities to close the gap between public health, ecosystem conservation and sustainability.*"

The basis of the study is whether snakebite should be considered a zoonotic disease. Perhaps more weightage should have been given to bites, circumstances, envenomed cases and medical case history of envenomed patients.

R: Yes, agreed that such information is critical to ultimately attribute risk levels to occupation; however, such data do not exist in a way that we can incorporate into the model, hence interpretation has to be inferential on the basis that farming and poverty are globally recognised risk factors for bites and their health consequences for victims. Similarly, land cover and occupation correlate, which allowed us to infer that parameter estimates for land cover represent occupational risks (line 129, for instance).

What the authors intend to justify should be succinctly and clearly stated. Many of the statements in the draft are self contradictory and confusing. The analogy regarding low envenoming by *D russelii* and *Naja naja* when abundantly found is very different from what is documented regarding both the species in scientific studies and field observations.

R: Thank you. In the case of *D russelii* and *Naja naja*, we argue that our estimates explain spatial heterogeneity, and given that both species are widespread across Sri Lanka, they have relatively low explanatory power of the observed spatial heterogeneity of snakebite risk. This means that while they are biologically relevant, contributing with a large number of bites, the numbers attributable to them do not vary across space as much as other species' bites (*Bungarus caeruleus* and *Hypnale spp.*, for instance). We have explained the above in lines 452-470:

"*… . However the lack of statistical significance of the estimates (credible intervals contain zero, Table 3), reflect a lack of explanatory power of the spatial heterogeneity, not a lack of biological relevance [53]. Therefore, the contradictory estimates from an epidemiological basis result from the higher envenoming incidence in east than west Sri Lanka (Figure 4) because both D. russelli and N. Naja are nearly equally abundant on both sides [2][28]. An alternative ecological explanation for the estimated negative effects for these two very medically-relevant species could arise if the predicted abundances of N. naja and D. russelli actually serve as proxies for the abundances of non-envenoming species. Abundance surrogacy is relatively common among habitat generalists [54] and non-envenoming snakes are commonly involved in snakebite cases [55], resulting in decreasing risk with the abundance of those non-medically relevant species. In conclusion, these population-level uncertainties related to the identity of the biting snakes, adequate clinical management should be based on expertise and careful clinical evaluation of the envenoming disease.*"

Results

Reviewer #1

see above/ I do not want to comment

Reviewer #2

The models of magnitude, distribution of snakebites, and envenoming incidence rates were well explained by the author, and this part has matched the analysis plan. The way the results were presented was clear. The author has clearly indicated how the analysis was done and what kind of models were used in the study.

R: Thank you

Reviewer #3

Accept

R: Thank you

Reviewer #4

What the authors intend to justify should be succinctly and clearly stated. Many of the statements in the draft are self contradictory and confusing. The analogy regarding low envenoming by *D russelii* and *Naja naja* when abundantly found is very different from what is documented regarding both the species in scientific studies and field observations.

R: This comment is the same as the one in the Methods section (see response above)

Given my background, I was not able to appropriately review the formulae for Functional Relationships for human snake contacts.

R: We understand, but thank you for the previous comments which helped make our explanation more biologically robust and coherent.

Conclusions

Reviewer #1

Data-based/Evidence-based medicine is absolutely indispensable for an effective disease control, so providing data, as this study does, is absolutely needed and is especially useful when planning interventions/prevention programs or treating a snakebite without informations regarding the snake. Nevertheless, every snakebite patient still should be assessed/examined/interrogated individually and treated individually. Not every single snake might have read the book when, where an whom to bite, so there might emerge danger when treating cases "prejudiced" by statistics. In my personal opinion, this limit should be mentioned with a few words in the discussion.

R: We agree with the reviewer. We certainly do not intend to convey the message that snakebite is a completely deterministic process, and to make this clear we have added the following to the discussion in lines 469-471 (track changes version):

"*... . In conclusion, given these population-level uncertainties related to the identity of the biting snakes, adequate clinical management in case of a bite should of course be based on expertise and careful clinical evaluation of the envenoming disease.*"

Reviewer #2

By describing the formulas, the author explained how mathematical modelling can be fit into epidemiological tools for snakebites. The author considered all the possible factors from both the snake and human sides. The snake sides included different species and their envenoming nature, as well as the aggressiveness of the snake. Human factors include social status, occupation, and culture. With these features incorporated, the conclusion has provided a good sketch of generalised epidemiological models for snakebites.

R: Thank you

Reviewer #3

Accept

R: Thank you

Reviewer #4

The basis of the study is whether snakebite should be considered a zoonotic disease. Perhaps more weightage should have been given to bites, circumstances, envenomed cases and medical case history of envenomed patients to justify why it should be categorized differently. Instead more effort has gone in snake distribution and contradictory observations of the same species in East and West Sri Lanka.

R: Following Reviewer 1's comment, we now address the novelty of the study as the application of modelling zoonoses to snakebites, which is reflected in our new title. We agree that detailed data is helpful, however such data does not exist throughout the study region. We believe snake ecology is as important as medical data as the estimation of snake distribution and abundance directly contributes to potential snakebite incidence (i.e., if there is no snake, there won't be any snakebites) and therefore provides a novel source of data that could be leveraged for improved management.

As for the "contradictory observations of the same species...", this has been addressed above and changes to the manuscript appear in lines 451-470 of the discussion.

Editorial and Data Presentation Modifications?

Reviewer #1:

(No Response)

Reviewer #2

The study is well-written and I would accept it.

R: Thank you.

Reviewer #3

Minor revisions:

(Line 106 and 107) Inconsistent use of the terms: “snakebite”, “snake bite”.

R: Done

(Line 108) Consider replacing line with “In future studies would allow us”.

R: Done

Typos in lines: 302 (“vaulues”) Figure 3 of supplementary material: (“bundances”)

R: Done

Format issues: Unequal size of text in Table 2.

R: Done, changed text size to 12 pt.

Table 3. For clarity, maybe explain abbreviations of “H. spp” - Hypnale spp and “T.trig.” with T. trigonocephalus on the Table 2 description.

R: Done.

Reviewer #4

(No Response)

Summary and General Comments

Reviewer #1

see above/ I do not want to comment

Reviewer #2

Many formulas were used in the study, but the author was able to explain them thoroughly. The data presentation and figures are clearly tied to the outcomes.

R: Thank you.

Reviewer #3

General comments

It would be interesting to have more information on how some other ecologic factors had an impact on the model. On lines 324-327, for example the effect of human population on snake abundance is

described by land cover classes. Yet it's important to describe that other species exist in varying degree in such land cover classes and not only humans and that these other species may also have an effect by being predators or prey of the vertebrate host of interest (snakes).

R: Yes, we agree with the observation. We would love to include even more interactions within the ecological community such as predators or competitors of biting species (other than humans); however at present data is strongly limiting. Similarly, data are very scarce on how snake populations respond to human presence, hence any effects of humans on snakes we could detect are related, as you point to land cover, but also as we point in the manuscript, to increasing human population density.

The authors don't seem to mention or include in the model the effect of seasonal variability, a well-known factor in snake-human interactions. They seem to address this only somewhat tangentially by considering the alignment of human-snake activity periods. If in fact was part of the analysis, this may warrant some clarification.

R: While seasonality is an important part of snake-human interactions that we have included in some of our other snakebite studies (e.g., Goldstein et al., 2021), we only focused on annual trends that group or integrate all seasons because at the time these analyses were conducted, country-wide spatio-temporal estimates were not available (e. g. (Ediriweera et al. 2021)). We have added the following sentence to the methods section to clarify (line 124-125, track changes version):

"*... continuous to discrete time, representing annual trends without seasonality, and estimated ...*"

Additional information would be useful to better understand some parameters. Why is "Tea" a land cover class, is it the main crop in the region? Also, it is not very clear how they determined what the most "medically relevant species are" (incidence of accidents, severity of cases, both?)

R: Yes, "Tea" is a main crop in numerous regions of Sri Lanka. Regarding medical relevance, species correspond to all species of the families Viperidae and Elapidae found in Sri Lanka, and exclude all other snake species, as these two families have and use front fangs to deliver venom. We now provide this information in line 139 of the Methods section:

"*... venomous snakes of Sri Lanka (three elapids and four vipers), estimated ...*"

Specific comments / minor issues

(Line 117-118) While it's quite understandable that they would exclude from the model a frequency-dependent analysis, it could be still applicable as some snakes after biting (not only humans but also prey), can become venom depleted for a few days, which, if following a zoonosis model, might be something to consider.

R: Yes, this is a good point, we do already assume that dry bites represent a fraction of total bites. However there is very little data around the potential frequency of venom injection between species. We now address this in the methods section, line 120:

"*… . Frequency dependence might function for snakebites in the presence of dry bites, such as occurs when venom glands are depleted [20]. However, its causes are still poorly understood to be incorporated in a process-based model. …*"

(Lines 140-141) The authors mention that the reservoir hosts raster was estimated with data from a 10 to 11-month period. This would be important to clarify as snake activity varies greatly throughout the year and the lack of information of even that month might be important.

R: Apologies, our description was insufficient and caused this confusion - snake abundance estimates for this study were based on decades of occurrence data. We now provide this information in line 141 of the Methods section:

"*… . Species records to build abundance models span several decades of fieldwork across the island …*"

(Lines 194-195) Statistical Analysis Software should be informed in the methodology according to convention. (Seems to be R and its packages (JAGS) and on line 279 (NIMBLE). Also, there is no information for the type of geographical information software used.

R: Yes, we used JAGS and NIMBLE via R. We also used R to manage geographical information via the raster and rgdal packages. We have now included the requested information to the methods section (line 198).

Reviewer #4

Page 8, Line 64 to Page 9, line 72 : Here we have considered only venomous snakes and envenoming scenarios. There are plenty of non-venomous snakes with scavenging habit which ensures presence of deadly pathogens in their saliva. Eg Fowlea piscator can inflict nasty wounds that can fester if proper and timely wound management is not done.

R: Infections are indeed a common complication in bite sites of many reptiles; however, these being real infections are outside the scope of our analyses which refer to snakebite envenoming.

Page 9, line 75 : you may want to include the following - For instance, working and living in an agrarian setting could increase spatio-temporal alignment; poverty could limit the use of protective clothing and also exposure to snakebites due to fragile dwellings made of mud and locally found materials that can influence a high probability of envenoming inside houses.

R: Done.

Given my background, I was not able to appropriately review the formulae for Functional Relationships for human snake contacts.

R: Not a problem, thank you for your important feedback regarding medical and epidemiological aspects.

Page 19, Table 1. Please do a spell check of "Transmission"

R: Done

Page 25, Line 383: Can we swap the word "aggressiveness" to "circumstances of the bite"

R: We believe the proposed change does not reflect the intended meaning, and have added the following:

"*... biological characteristics of the different species, like aggressiveness and severity of the envenoming illness* ***produced by its bite***. *...*"

Page 27. Finally the negative coefficients estimated for D. russelii and N. naja (Table 3) indicate that the probability that a bite results in envenoming decreases when these species are more abundant.

R: Yes that is the straightforward interpretation; however as we point out, coefficients were not significantly different from zero, which means that *D. russellii* and *N. naja* do not explain spatial heterogeneity for the reasons explained above.

Question: Would like to defer on the above analogy. Is this based on any scientific evidence?

R: The only existing evidence is the present study; however, as with all statistical analyses they have to be interpreted as we clarify in the previous point.

Page 26, Line 397 : In particular, snake occurrence records and behavioural trait data were the raw material upon which we built our model

R: This paragraph was perhaps meant to be part of the previous one. In which case, yes, snake abundance patterns were estimated using point data (geographical coordinates of species' individuals).

Question: Is the man-snake contact data used from anecdotal documentation or information from a repository maintained by an App or local govt department? What is the source from where the data was picked up for the study?

R: Human-snake contact rates were estimated by us with the Bayesian model simulating parameter values with a pre-specified statistical distribution until we obtained values that consistently explained incidence rates estimated by Ediriweera et al. (2016). The rates we report have not been published previously, nor is there an existing simple and fast way to estimate them.

The basis of the study is whether snakebite should be considered a zoonotic disease. Perhaps more weightage should have been given to bites, circumstances, envenomed cases and medical case history of envenomed patients.

R: This comment is repeated, please see response above.

Page 27, line 430 : 3) Why do highly medically-important species' decrease the probability that bites are envenoming?

R: Explanation of this aspect is given above

Question : Not sure what the above means. There are snakes that are highly venomous with very few reported bites. But there are also countries with snake distribution of highly venomous snakes that are responsible for thousands of deaths annually. *D. russelii* and *Naja naja* envenoming related deaths and disabilities is quiet high in South Asian countries.

R: Yes this is true, however, as we suggest, our coefficient estimates are not significantly different from zero, which means that neither Russel's viper nor cobra explain spatial heterogeneity. We are not saying these species are not of high medical importance (please see above response to a similar comment).

Page 27 Line 436 : Finally the negative coefficients estimated for *D. russelii* and *N. naja* (Table 3) indicate that the probability that a bite results in envenoming decreases when these species are more abundant. Such a negative relationship is unexpected as both are considered among the primary cause of snakebites in Sri Lanka

The above finding has no logic.

R: Please see explanations above.

Pg 27, line 442 : Hence their abundances cannot explain the east vs west spatial heterogeneity of envenomings, making estimation of their effects difficult with the methods used here

What the authors intend to justify should be succinctly and clearly stated. The above statement is confusing.

R: Agreed. We have now included a more concise and straightforward explanation in lines 442-471 (track changes version) of the discussion (see document with track changes as these are relatively long to list here).

References

Eyal Goldstein, Joseph J. Erinjery, Gerardo Martín, Anuradhani Kasturiratne, Dileepa Senajith Ediriweera, , H. Janaka de Silva, David G. Lalloo, Kris A. Murray and Takuya Iwamura. 2021. Integrating human behavior and snake ecology with agent-based models to predict snakebite in high risk landscapes. *PLoS Neglected Tropical Diseases* 15(1): 1-20. https://dx.plos.org/10.1371/journal.pntd.0009047.

Ediriweera, Dileepa Senajith, Anuradhani Kasthuriratne, Arunasalam Pathmeswaran, Nipul Kithsiri Gunawardene, Shaluka Francis Jayamanne, Kris Murray, Takuya Iwamura, et al. 2021. 'Evaluating Spatiotemporal Dynamics of Snakebite in Sri Lanka: Monthly Incidence Mapping from a National Representative Survey Sample'. *PLOS Neglected Tropical Diseases* 15 (6): e0009447. https://doi.org/10.1371/journal.pntd.0009447.

Ediriweera, Dileepa Senajith, Anuradhani Kasturiratne, Arunasalam Pathmeswaran, Nipul Kithsiri Gunawardena, Buddhika Asiri Wijayawickrama, Shaluka Francis Jayamanne, Geoffrey Kennedy Isbister, et al. 2016. 'Mapping the Risk of Snakebite in Sri Lanka - A National Survey with Geospatial Analysis'. *PLoS Neglected Tropical Diseases* 10 (7): 1–14. https://doi.org/10.1371/journal.pntd.0004813.
