# Supplementary material for: A mechanistic model of snakebite as a zoonosis: Envenoming incidence is driven by snake ecology, socioeconomics and its impacts on snakes
Source: PLoS Negl Trop Dis. 2022 May 12;16(5):e0009867. doi: 10.1371/journal.pntd.0009867 (PMC9129040; doi:10.1371/journal.pntd.0009867)
Supplement: Attachment — Submitted filename: PNTD-D-21-01445_Reviewer comments.docx [file pntd.0009867.s007.docx]

Snakebite to be considered a zoonotic disease is a very important study and perspective. The only concern is the data being referred is unclear and more than a decade old. Urbanization, deforestation, increase in population and climate change related untimely season changes have caused environmental changes affecting flora and fauna in many places. Urbanization juxtaposing with farming activities and irresponsible garbage dumping seem to have resulted in a higher number of man-animal conflict cases in countries in Southeast Asia, especially where snake distribution thrives on prey base found abundantly close to human habitations.

Page 8, Line 64 to Page 9, line 72 : Here we have considered only venomous snakes and envenoming scenarios. There are plenty of non-venomous snakes with scavenging habit which ensures presence of deadly pathogens in their saliva. Eg Fowlea piscator can inflict nasty wounds that can fester if proper and timely wound management is not done.

Page 9, line 75 : you may want to include the following - For instance, working and living in an agrarian setting could increase spatio-temporal alignment; poverty could limit the use of protective clothing and also exposure to snakebites due to **fragile dwellings made of mud and locally found materials** that can influence a high probability of envenoming inside houses.

Given my background, I was not able to appropriately review the formulae for Functional Relationships for human snake contacts.

Page 19, Table 1. Please do a spell check of “Transmission”

Page 25, Line 383 : Can we swap the word “aggressiveness” to “circumstances of the bite”

Page 27. Finally the negative coefficients estimated for *D. russelii* and *N. naja* (Table 3) indicate that the probability that a bite results in envenoming decreases when these species are more abundant.

Question: Would like to defer on the above analogy. Is this based on any scientific evidence?

Page 26, Line 397 : In particular, snake occurrence records and behavioural trait data were the raw material upon which we built our model

Question: Is the man-snake contact data used from anecdotal documentation or information from a repository maintained by an App or local govt department? What is the source from where the data was picked up for the study?

The basis of the study is whether snakebite should be considered a zoonotic disease. Perhaps more weightage should have been given to bites, circumstances, envenomed cases and medical case history of envenomed patients.

Page 27, line 430 : 3) Why do highly medically-important species’ decrease the probability

that bites are envenoming?

Question : Not sure what the above means. There are snakes that are highly venomous with very few reported bites. But there are also countries with snake distribution of highly venomous snakes that are responsible for thousands of deaths annually. D. russelii and Naja naja envenoming related deaths and disabilities is quiet high in South Asian countries.

Page 27 Line 436 : Finally the negative coefficients estimated for *D. russelii* and *N. naja* (Table 3) indicate that the probability that a bite results in envenoming decreases when these species are more abundant. Such a negative relationship is unexpected as both are considered among the primary cause of snakebites in Sri Lanka

The above finding has no logic.

Pg 27, line 442 : Hence their abundances cannot explain the east vs west spatial heterogeneity of envenomings, making estimation of their effects difficult with the methods used here

What the authors intend to justify should be succinctly and clearly stated. The above statement is confusing.
